# Mitochondrial disease, mitophagy, and cellular distress in methylmalonic acidemia

**DOI:** 10.1007/s00018-021-03934-3

**Published:** 2021-09-15

**Authors:** Alessandro Luciani, Matthew C. S. Denley, Larissa P. Govers, Vincenzo Sorrentino, D. Sean Froese

**Affiliations:** 1 Mechanisms of Inherited Kidney Diseases Group, Institute of Physiology, University of Zurich, 8032 Zurich, Switzerland; 2 Division of Metabolism and Children’s Research Center, University Children’s Hospital Zurich, University of Zurich, 8032 Zurich, Switzerland; 3 Department of Musculo-Skeletal Health, Nestlé Institute of Health Sciences, Nestlé Research, 1015 Lausanne, Switzerland

**Keywords:** Cell damage, Inherited metabolic diseases, Metabolism, Mitochondria, Mitophagy, Oxidative stress

## Abstract

Mitochondria—the intracellular powerhouse in which nutrients are converted into energy in the form of ATP or heat—are highly dynamic, double-membraned organelles that harness a plethora of cellular functions that sustain energy metabolism and homeostasis. Exciting new discoveries now indicate that the maintenance of this ever changing and functionally pleiotropic organelle is particularly relevant in terminally differentiated cells that are highly dependent on aerobic metabolism. Given the central role in maintaining metabolic and physiological homeostasis, dysregulation of the mitochondrial network might therefore confer a potentially devastating vulnerability to high-energy requiring cell types, contributing to a broad variety of hereditary and acquired diseases. In this Review, we highlight the biological functions of mitochondria-localized enzymes from the perspective of understanding—and potentially reversing—the pathophysiology of inherited disorders affecting the homeostasis of the mitochondrial network and cellular metabolism. Using methylmalonic acidemia as a paradigm of complex mitochondrial dysfunction, we discuss how mitochondrial directed-signaling circuitries govern the homeostasis and physiology of specialized cell types and how these may be disturbed in disease. This Review also provides a critical analysis of affected tissues, potential molecular mechanisms, and novel cellular and animal models of methylmalonic acidemia which are being used to develop new therapeutic options for this disease. These insights might ultimately lead to new therapeutics, not only for methylmalonic acidemia, but also for other currently intractable mitochondrial diseases, potentially transforming our ability to regulate homeostasis and health.

## Introduction

Mitochondria are complex dynamic organelles that take the pilot’s seat in the biology of most eukaryotic cells by playing a key part in catabolic and anabolic metabolism, hence guiding cell-wide programs for cellular growth and organismal homeostasis. Ever since its initial description by Altmann [1] and Benda [2] in the 1890s, the mitochondrion has come to be known as a double-membrane-enclosed cytoplasmic organelle responsible for the generation of cellular energy through oxidative phosphorylation (OXPHOS) [3]. In parallel with its role in the production of cellular energy in the form of ATP, this highly dynamic organelle regulates intracellular calcium [4–6] and redox homeostasis [7, 8]. It also acts as a cardinal platform to coordinate molecular circuitries, toggling the balance between cell survival and regulated cell death pathways in response to changes in the surrounding microenvironment or stress conditions, hence driving nearly every aspect of cell differentiation, fate, and function [9]. Given the central role in preserving cellular and physiological homeostasis, imbalances in mitochondrial function thus pose a potentially devastating threat to many different cell types, fuelling pathologies associated with ageing, neurodegeneration, metabolic disease, and cancer [10–13].

Over the last two decades, studies of rare inherited genetic diseases, in combination with advances in technology and foundational genomic resources enabled by the high-throughput omics era, have provided novel insights into fundamental principles governing the contribution of mitochondria to cellular homeostasis and physiology. Through converging approaches, these pathway paradigms have captured a holistic understanding of the pathogenesis of other common human disorders in which mitochondrial dysfunction has been implicated as a secondary event in the progression of the disease. Furthermore, genome-wide association studies of urinary metabolites have identified the association between common sequence variants within or close to the genes encoding enzymes and transport proteins that enrich the mitochondrial matrix—the subcellular compartment in which many detoxification reactions and fatty acid and amino acid metabolism occur—and kidney-related traits (i.e. urine albumin-to-creatinine ratio and estimated glomerular filtration rate [13]) or different disease states, including chronic kidney disease [14]. These large-scale and multi-omics approaches thus support the fundamental role of mitochondria in organizing cellular homeostasis and organismal physiology, and its contribution to disease risk within the population.

In this review, we discuss the role of the mitochondria, not only as an energetic powerhouse, but also as a hub for signaling and homeostasis pathways and delve into the breakdown and removal of dysfunctional mitochondria through degradative processes. We further investigate diseases specifically affecting the mitochondria and take the dysfunction of the mitochondrial matrix residing metabolic enzyme methylmalonyl-coenzyme A mutase (MMUT) as a paradigm of mitochondrial disease. We describe the multiple roles of this enzyme in regulating the mitochondrial network and possible molecular and cellular pathways through which the loss-of-function mutations in *MMUT* might lead to mitochondrial distress, resulting in organ dysfunction primarily affecting the brain and kidney, and ultimately causing life -threatening manifestations. This Review also places a special emphasis on emerging advances in (patient-derived) cell-based and novel preclinical animal-based models that are empowering the development of small molecule-based drug discovery and screening programs. In the concluding section, we highlight regulatory pathways targeting cellular adversities linked to MMUT dysfunction and potential targets for therapeutically treating this life-threatening disease.

## Mitochondria: from structure to physiology to organelle quality control

### Structure

Mitochondria, whose name was coined by Carl Benda [2] in 1898 by combining the Greek words “mitos” (meaning thread) and “chondrion” (meaning granule), can be either found as isolated organelles or form large networks. They can have different morphological shapes and ultrastructural organization and distribute unevenly in the cytoplasm through specialized transport and positioning to adequately respond to changes in microenvironmental cues [15].

These highly dynamic and plastic organelles harbor multiple copies of their own bacterium-derived mitochondrial DNA (mtDNA) in the form of circular double-stranded DNA molecules. The mammalian mitochondria-derived proteome is composed of approximately 1500 proteins [16, 17] nearly all of which are encoded by nuclear DNA, translated in the cytosol, and delivered to mitochondrial sub-compartments through regulated import systems, processing, and assembly. By contrast, mtDNA only codifies approximately 1% of the mitochondrial proteome; these latter proteins being components of OXPHOS complexes [18]. Moreover, mtDNA mutations, whose rate is higher than the nuclear genome, can lead to metabolic dyshomeostasis, with defects in cellular energy production and tissue dysfunction, as testified by the identification of a large number of mitochondrial diseases [19]. Concomitantly, mutations in mtDNA might also accumulate during aging, wreaking havoc, directly or indirectly, on mitochondrial health and functionality, eventually contributing to age-induced adversities of a multitude of pathologies [18].

**Fig. 1:**
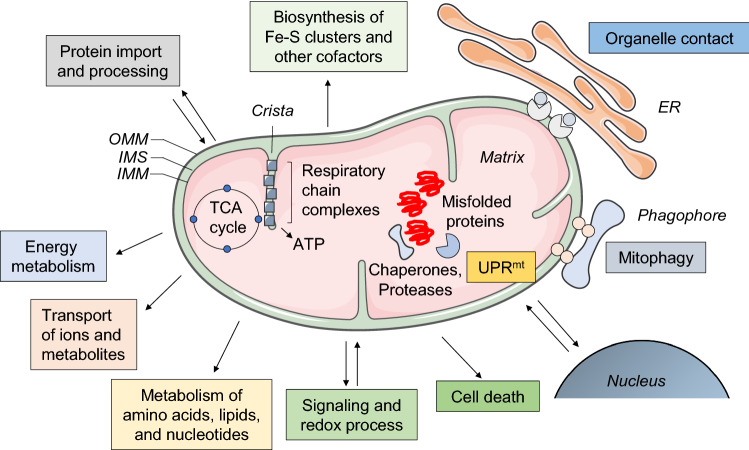
**Mitochondria and their functions**. Mitochondria consist of four compartments: outer membrane (OMM), intermembrane space (IMS), inner membrane (IMM), and matrix. Mitochondrial proteins and complexes are involved in energy metabolism with respiration and biosynthesis of ATP; metabolism of amino acids, lipids, and nucleotides; biosynthesis of iron sulfur (Fe‒S) clusters and other cofactors; transport of ions and metabolites; protein import and processing; signaling and redox homeostasis; membrane architecture and dynamics, and interorganelle communication by establishing contact sites; and quality control and degradation processes, and cell death pathways

Mitochondria are composed of two structurally and functionally different membranes (Fig. 1), described as the outer mitochondrial membrane (OMM) and the inner mitochondrial membrane (IMM). The smooth and permeable OMM acts as a platform for the transmission and decoding of signaling cascades into the mitochondria. Beyond its primordial function as a barrier and its latter evolution into a signaling hub, the OMM also establishes a networks of physical and functional interactions with other intracellular moieties (Fig. 1)—such as the endoplasmic reticulum (ER), endosome and lysosome, peroxisome, plasma membrane, and lipid droplets—through the formation of membrane contact sites where the exchange of ions, metabolites, and other molecules can take place. Further details on the composition, dynamics, identity, and specific functions of membrane contact sites have been exhaustively reviewed elsewhere [20, 21]. By contrast, the IMM, which surrounds the mitochondrial matrix, is more intimately involved in bioenergetics. The IMM can be further divided into two sub-compartments: the inner boundary membrane and the inner membrane cristae, the latter of which are deeply convoluted invaginations that harbor the machinery required for mitochondrial respiration, including the mitochondrial respiratory chain and F_1_F_0_-ATP synthase [22, 23], ensuring an adequate ATP conversion and energy metabolism (Fig. 1). Altogether, the maintenance of the mitochondrial network structure and integrity is key to performing a wide range of metabolic pathways and signaling cascades that sustain cellular and physiological homeostasis.

### Diverse functions of mitochondria

One of the main functional roles attributed to mitochondria is the ability to coordinate ATP production and energy conversion. Typically, mitochondria comprise the respiratory chain complexes and F_1_F_0_ ATP synthase, which are located to the inner membrane cristae, and a broad string of nutrient transporters and channels for metabolites and other molecules, which are mostly situated within the matrix and the IMM [24, 25]. Electrons from the oxidation reactions of metabolites—such as tricarboxylic acid cycle intermediates, amino acids, lipids, and nucleotides—sustain the respiratory chain, ultimately triggering an electrochemical gradient that pumps protons out of the mitochondrial matrix into the intermembrane space [26]. The resulting proton gradient drives the conversion of ADP to ATP through F_1_F_0_ ATP synthase in the process of OXPHOS and enables the transport of precursor proteins and metabolites across the IMM [26]. Under aerobic conditions, this process is responsible for generating the majority of cellular energy and participates in nearly every aspect of metabolic functions.

Beyond its role in energy production and cellular metabolism, the mitochondrial network (Fig. 1) is required for fatty acid, amino acid, and nucleotide metabolism [27], iron–sulfur cluster and cofactor biogenesis [28], and import and processing of precursor proteins that are synthetized on cytosolic ribosomes [29]. Furthermore, mitochondria can serve as signaling platforms that regulate programmed cell death and innate immunity [12, 30]. For instance, virus infection can activate mitochondrial antiviral signaling proteins, which localize to the OMM and trigger the production of cytokines to counteract infection [31].

Mitochondria also have major roles in sequestering and releasing calcium ions, hence regulating the cellular stores of these versatile signaling molecules. Owing to their intrinsically dynamic nature and the formation of membrane contact sites with the ER, mitochondria utilize calcium transporters [4, 5] located at the IMM to control cellular calcium homeostasis in response to contextual signals, ultimately safeguarding a vast array of biological processes and cellular functions. Altogether, the maintenance of a healthy and functional mitochondrial network is particularly critical for the viability of eukaryotic cells as these organelles perform essential functions in bioenergetics, metabolism, and signaling.

### Mechanisms of mitochondrial quality control

Mammalian cells have evolved elaborated quality control and surveillance systems to cope with perturbations of homeostasis [32, 33]. For instance, the accumulation of misfolded proteins owing to increased mitochondrial ROS or extra-mitochondrial proteotoxicity or alterations of the respiratory complexes or mitochondrial translation [34, 35]can activate an evolutionarily conserved, transcriptional stress response aptly coined as the mitochondrial unfolded protein response (UPR^mt^; Fig. 1), part of a larger cell-wide stress response. Within the mitochondria, the UPR^mt^ promotes the repair and recovery of mitochondrial network proteostasis, thereby counteracting proteotoxic stress and ensuring the maintenance of mitochondrial and cellular functions [27]. In *Caenorhabditis elegans*, as part of the UPR^mt^, an activating transcription factor associated with stress (ATFS‒1 [36]), which is normally imported into healthy mitochondria through its mitochondrial protein import sequence and degraded, is instead targeted to the nucleus to trigger adaptive responses that enable the cell to cope with mitochondrial distress [37]. This results in the accumulation of nuclear transcripts that stimulate the recovery of OXPHOS complexes [38, 39], re‒establish mitochondrial proteostasis by upregulating chaperones and proteases, and detoxify ROS [40, 41], ultimately restoring mitochondrial proteostasis and homeostasis. However, mammalian studies paint a different picture that implies a process called the integrated stress response, which lowers overall protein production while increasing the abundance of transcriptional factors [40]. Respective findings by Guo et al., [42] and Fessler et al., [43] suggest that mitochondrial dysfunction causes the protein OMA1, which is located on the IMM, to cleave the protein DELE1, a fragment of which enters the cytosol and binds to the enzyme HRI, subsequently activating it. HRI adds a phosphate group to the protein eIF2α, and this phosphorylation slows the synthesis of most cellular proteins from messenger RNA, mediated by the ribosome complex, but promotes the production of the transcription factors ATF4, ATF5 and CHOP. Treatments that activate the UPR^mt^ have been shown to ameliorate mitochondrial function in amyloid-β proteotoxic diseases, such as Alzheimer’s disease [44], age-associated amyloidosis [45], and kidney injury [46]. Conversely, tuning the chronic integrated stress response activation might potentially rescue synaptic plasticity loss [47], and reverse age-related cognitive decline [48], and sepsis-induced kidney injury [49].

Recent studies have indicated that similar forms of mitochondrial dysfunction, including damage by paraquat, mtDNA mutations, and misfolded protein accumulation in the matrix, might activate UPR^mt^ as well as other quality control and surveillance systems. For example, mitochondrial fusion proteins—such as the dynamin-like mitofusin 1(MFN1), MFN2 and OPA1—combat mitochondrial stress by fusing damaged and/or dysfunctional mitochondria with healthy mitochondria [21, 50]. Alternatively, proteins involved in mitochondrial fission—including the cytosolic dynamin-like protein DRP1 and its outer mitochondrial membrane partners—segregate exhausted parts of the mitochondrial network, which are then selectively removed by lysosome-directed degradation pathways [51]. This fascinating interplay between fusion and fission membrane dynamics and mitochondrial quality control mechanisms has been extensively discussed elsewhere [52, 53]. Here, we will emphasize the signaling cascades regulating the mechanisms of mitochondrial elimination by autophagy-lysosome degradation systems.

An essential facet of quality control mechanisms is the activation of (largely selective) degradative cascades that enable cells to dispose of exhausted and/or potentially harmful mitochondria. This may occur through the dynamic and self-regulated process aptly coined as mitochondrial autophagy/mitophagy (Fig. 1), which results in the engulfment of cellular constituents by a double-membrane structure called an autophagosome [33, 54–57]. In functional mitochondria, the phosphatase and tensin homologue (PTEN)-induced kinase 1 (PINK1) is transported into the mitochondrial matrix, where it is processed and cleaved [52, 58, 59] by matrix processing peptidase (MPP) and presenilins-associated rhomboid-like protein (PARL). As a result, the truncated form of PINK1 is released into the cytosol and degraded by the ubiquitin–proteasome system [60]. Conversely, dissipation of the mitochondrial membrane potential triggers the stabilization of PINK1, facilitating its accumulation on the OMM [61]. This leads to activation of the E3 ubiquitin ligase Parkin, which triggers the ubiquitination of other OMM proteins, culminating in the recruitment of autophagy initiating factors [60, 61] and engulfment of damaged mitochondria within autophagy-lysosome degradation pathways.

Beyond its role in organelle quality control and surveillance systems, Parkin has also been found to induce the activation of the transcriptional co-activator peroxisome proliferator-activated receptor gamma co-activator 1 alpha (PGC1α)—a key regulator of mitochondrial biogenesis and energy metabolism—through the ubiquitination of Parkin-interacting substrate [62, 63]. Upon mitochondrial damage, the PINK1-directed recruitment and activation of Parkin at the OMM can trigger the ubiquitin/proteasome system (UPS)-mediated degradation of PARIS—a transcriptional repressor of PGC1—ultimately stimulating mitochondrial biogenesis and function [62, 63]. In a similar vein, the modulation of PGC1α-elicited mitochondrial biogenesis triggered by changes in sirtuin and NAD^+^metabolism [64], oxidative damage or environmental stress [65, 66], or during exercise [67], seems to trigger PINK1/Parkin-dependent turnover of mitochondria in different tissues and model organisms. Thus, an intimate cross-talk between signaling and degradation pathways safeguards the maintenance of a healthy mitochondrial repertoire, hence preserving its functions in cellular metabolism and organismal homeostasis.

Independent of ubiquitin-driven mitophagy, other OMM-associated mitophagy receptors, such as BNIP3 (BCL2/adenovirus E1B 19 kDa protein-interacting protein 3), NIX (NIP3-like protein X)/BNIP3L, and FUNDC1 (FUN14 domain-containing 1) target damaged and/or dysfunctional mitochondria to autophagosomes by directly promoting the binding of mitochondria with LC3 and GABA_A_-receptor-associated protein (GABARAP) through atypical or typical LC3-interacting motifs [68–71]. In a similar vein, cardiolipin—a phospholipid primarily synthesized and distributed along the IMM—may be redistributed to the OMM to interact with LC3-flagged autophagy cargos, initiating a signaling cascade that promotes the engulfment of damaged mitochondria by autophagosomes in response to mitochondrial damage [72], helping preserve cellular and physiological homeostasis.

In contrast to the wholesale mitophagy processes described above, a recently emerging piecemeal mitophagy mechanism of mitochondrial quality control has been described, involving the release of small vesicles excised from the mitochondria and hence aptly termed as mitochondrial-derived vesicles (MDVs [53]). These MDVs have been shown to engulf certain cargos—including outer and inner mitochondrial membrane and/or matrix residing proteins—and transfer them to the lysosome for degradation in an LC3- and p62/SQSTM1-dependent manner or to the peroxisome with as yet unclear consequences. Thus, MDVs might act as a first round of defense for mitochondria to remove damaged proteins, preventing the complete failure of the organelle. The diverse repertoire of cellular quality control pathways therefore seeks to ensure the maintenance of a functional mitochondrial network, ultimately sustaining energy metabolism, cellular and organismal homeostasis. Further insights into this fundamental pathway might ultimately lead to new treatments for mitochondrial disorders and other disease entities associated with mitochondrial dysfunction.

## Diseases affecting mitochondrial function

### Primary and secondary mitochondrial disorders

Given the crucial roles of the mitochondrial network in cellular energy production and homeostasis, it is not surprising that disorders which disrupt mitochondrial function are of grave consequence to the individual. When inherited, these disorders are often classified as primary or secondary mitochondrial diseases. Primary mitochondrial diseases include inherited disorders that disrupt OXPHOS or mitochondrial structure and function, such as abnormalities in the production of cofactors and vitamins, or other alterations in the TCA cycle and pyruvate metabolism [73]. Of the 1500 proteins estimated to participate in mitochondrial function and maintenance, nearly 400 have been reported to cause primary mitochondrial disease [74], including all 37 mitochondrial encoded genes. Altogether, primary mitochondrial disorders affect approximately 1:4300 births [75].

**Fig. 2:**
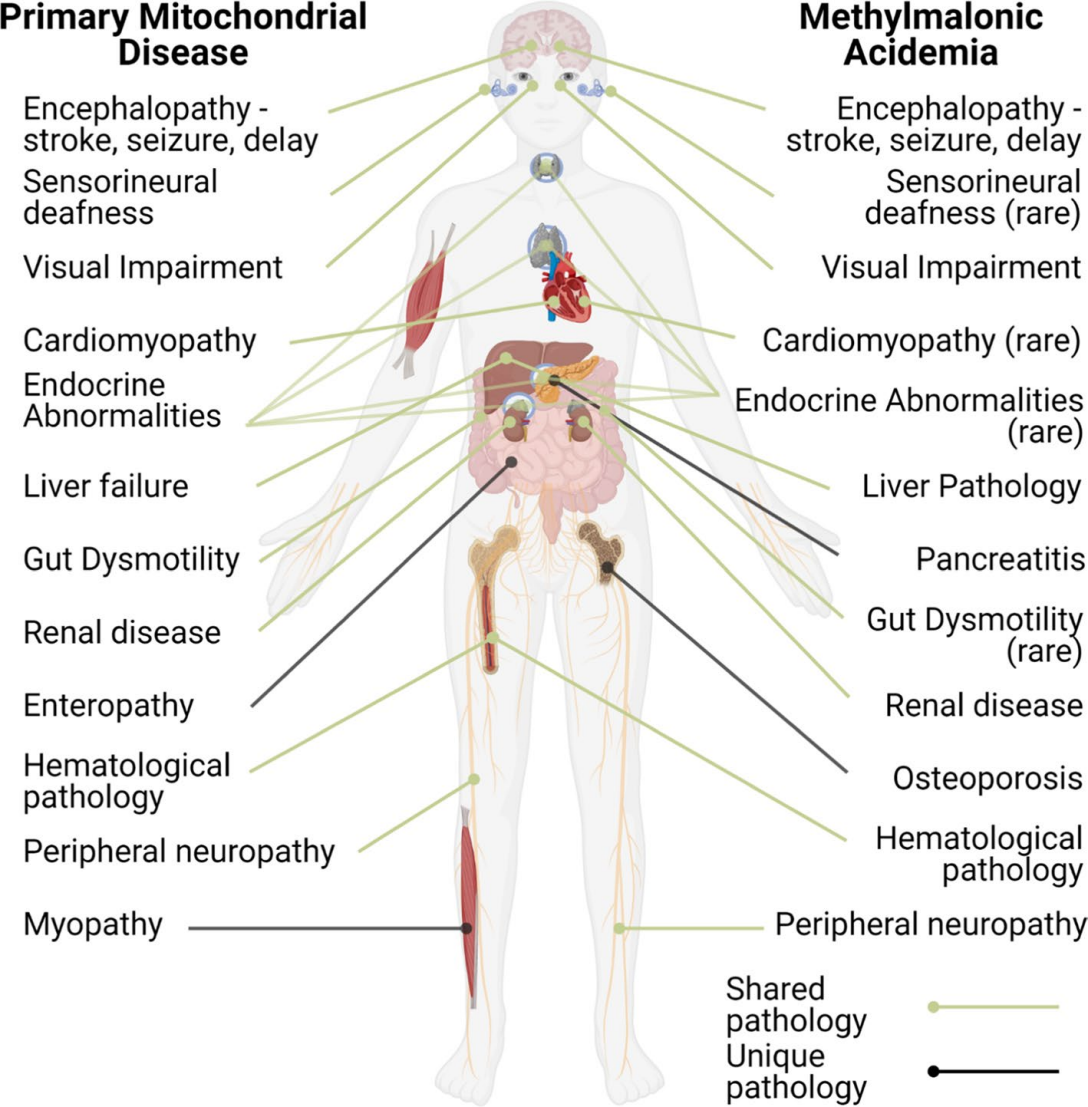
**Comparison of the clinical presentation of primary mitochondrial diseases and MMA.** Note the overlap of most clinical features, with some interesting exceptions, including cardio/myopathy (rare in MMA)

The heterogeneity of the processes disturbed by primary mitochondrial disorders is reflected by the phenotypic variability of the patients, in terms of tissues and organs affected, as well as by age of onset and presenting symptoms [73, 74]. Patients may exhibit manifestations in almost any tissue or organ, in a multi-systemic or tissue-specific manner, from the first days to weeks of life until after several decades [76]. Nevertheless, along with lactic acidosis, frequently identified symptoms involve encephalopathy, cardiomyopathy, renal insufficiency, and liver failure [73, 74] (Fig. 2). Clinical features in these disorders have been expertly reviewed elsewhere [74, 77].

A considerable body of evidence suggests that the mitochondrial stress responses triggered by a primary molecular defect in the organelle, and not defects of OXPHOS per se, are the major contributing factor to the clinical and biochemical features of mitochondrial disorders [3]. Such mechanisms, including the UPR^mt^, are likely to be activated by secondary mitochondrial diseases—i.e., disorders of genes/proteins not directly involved in OXPHOS or mitochondrial integrity, but which may indirectly compromise these essential processes through toxic metabolites or missing products.

A prototypical group of secondary mitochondrial disorders are the organic acidurias, a collection of diseases including branched-chain ketoaciduria, isovaleric aciduria, propionic aciduria and methylmalonic aciduria (MMA), whose disrupted pathways take place within the mitochondria [78]. In patients with these disorders, deficient activity of enzymes involved in energy producing, catabolic pathways, result in the accumulation of mono-, di- or tricarboxylic acids in the brain and other tissues as well as in the urine, plasma, and cerebrospinal fluid. Although the enzymes primarily disturbed in organic acidurias are not directly involved in OXPHOS, they do affect mitochondrial energy metabolism and mitochondrial homeostasis, whose dysfunction seems to have a major role in disease development [79]. This can also be seen from the clinical manifestations of these disorders, whereby frequent clinical symptoms include cardiomyopathy, optic atrophy and basal ganglia abnormalities, liver dysfunction and kidney failure, in analogy to primary mitochondrial disorders. All these manifestations, to varying degrees, have been found in MMA (Fig. 2), which can, therefore, be used as a prototypical model of (secondary) mitochondrial disease.

### MMA is a paradigm organic aciduria with secondary mitochondrial disease

#### Gene, mutations, and protein functions

MMA can be caused by a series of defects, most of which lead to deficiency of the enzyme methylmalonyl-CoA mutase (MMUT), with a combined prevalence estimated to be 1:50,000 [80]. Mutations in the *MMUT* gene account for approximately 50% of all cases of isolated MMA, and to date almost 300 inherited mutations have been described in *MMUT*, the majority of which are of the missense type and associated with the more severe phenotype that manifest in the absence of enzyme activity (mut^0^) [81]. Nevertheless, most mutations are private, and only a few mutations occur in several patients, including c.655A > T (p.Asn219Tyr), c.1106G > A (p.Arg369His), c.2080C > T (p.Arg694Trp) and c.2150G > T (p.Gly717Val) [81, 82].

MMUT catalyzes the reversible isomerization of l-methylmalonyl-CoA to succinyl-CoA—an intermediate of the tricarboxylic acid cycle that is further processed by succinyl-CoA ligase. This represents the culmination of propionyl-CoA catabolism, most of which derives from the breakdown of branched-chain amino acids and odd-chain fatty acids, although a significant proportion of propionyl-CoA may be derived from gut bacteria [83]. Propionyl-CoA is converted to d-methylmalonyl-CoA by the action of propionyl-CoA carboxylase, defects in which cause propionic aciduria—a disease that phenotypically and biochemically overlaps with MMA [84]. For use by MMUT, D-methylmalonyl-CoA is racemized to the L configuration by methylmalonyl-CoA epimerase (MCEE). For proper function, MMUT requires the vitamin B_12_ derived cofactor adenosylcobalamin. Therefore, defects in the genes *MMAA* [85], *MMAB* [86] and *MMADHC* [87], which are responsible for the synthesis or disposition of adenosylcobalamin, may also lead to MMUT deficiency and therefore MMA, while biochemically mild MMA can be associated with mutations in *MCEE* [88, 89], succinyl-CoA ligase (*SUCLG1* or *SUCLA2*) [90] or *ACSF3* (combined malonic and methylmalonic aciduria) [91, 92].

**Fig. 3:**
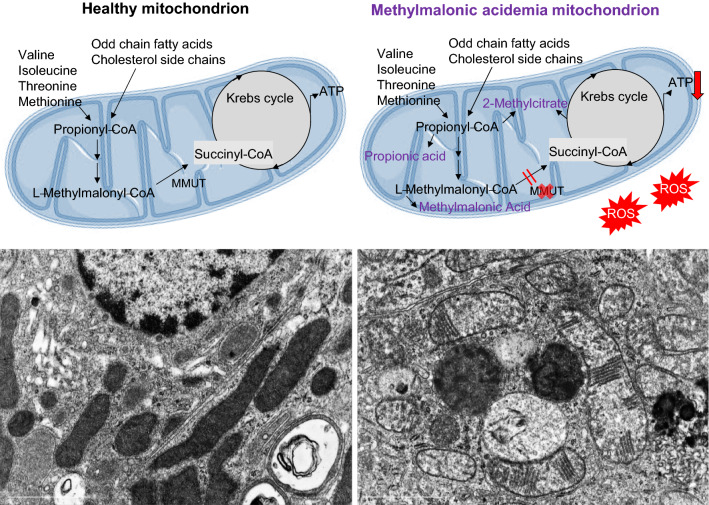
**Loss of the enzyme MMUT leads to accumulation of organic acids and mitochondrial abnormalities.** Inactivating mutations in the *MMUT* gene encoding the mitochondrial enzyme methylmalonyl-coenzyme A mutase, which mediates the terminal step of branched-chain amino acid and odd-chain lipid catabolism, results in the accumulation of metabolites (i.e., methylmalonic acid, propionic acid, and 2-methylcitric acid) and lack of anaplerosis. This triggers ultrastructural (i.e., presence of abnormal mitochondria with disorganized cristae) and/or functional (i.e., impaired mitochondrial energetics and redox homeostasis) mitochondrial alterations, ultimately causing severe organ dysfunctions that primarily affect brain, liver, and kidney. Electron micrographs courtesy of Francesca Diomedi (Adapted from Luciani et al*.* [130])

In MMA, organic acids in addition to the substrate methylmalonyl-CoA accumulate (Fig. 3), leading to secondary inhibition of several enzymes in TCA cycle and respiratory chain metabolism, the urea cycle, and mitochondrial transport [93]. This impairs mitochondrial respiration and ammonia metabolism, pathologies shared by other organic acidurias and primary mitochondrial disorders [94]. All of these are likely to play a role in the clinical presentation of this disease.

### Clinical features

The clinical presentation of MMA is often nonspecific and patients may present with acute or chronic symptoms at any age [84]. Nevertheless, a classical early-onset presentation in the short term may include metabolic decompensation with vomiting, feeding problems, metabolic acidosis and hyperammonemia with neurological deterioration including muscular hypotonia, irritability and lethargy, which in the most severe cases may lead to coma and death [95]. Current treatments, including intensive care, dietary modulation (protein restriction, precursor free amino acid supplements, tube-feeding) and pharmacotherapy (administration of cobalamin, carnitine and reduction of intestinal flora with antibiotics), have resulted in many patients surviving these initial crises [96, 97]. However, affected patients remain vulnerable to life-threatening metabolic decompensations, often show only slight improvement, and are not protected from long-term complications [96, 98]. The most striking long-term complications involve neurological symptoms and kidney manifestations [96, 99, 100]; while cardiomyopathy, a common feature of propionic aciduria [84, 101] and primary mitochondrial disorders, is not frequently found in MMA.

### Brain abnormalities

Neurological impairment in MMA can take many forms. In 52 children with MMA, the most common computed tomography (CT) and magnetic resonance (MR) brain sectional imaging findings were ventricular dilation (17 patients), cortical atrophy (15), periventricular white matter abnormality (12), thinning of the corpus callosum (8), subcortical white matter abnormality (6), cerebellar atrophy (4), basal ganglia calcification (3), and myelination delay (3) [102]. However, the clinical significance of many of these radiological findings is often unclear.

Neuropathological findings in white matter tracts, cerebral and cerebellar cortex are typically isolated to atrophy, reactive gliosis, spongiosis and hypomyelination [102–104]. In MMA, periventricular white matter abnormalities, which are defined as delayed myelination, occur early and may be partially reversible. Cortical atrophy in MMA is defined as ventricle and sulci widening and as with delayed myelination, early detection and treatment (e.g., before 2 years of age) may result in normalization after a period of time [102–106]. Seizures are also a recognized symptom of MMA which may be present at presentation or appear later [84, 96, 107].

Neurological impairment most commonly manifests as movement disorders, such as involuntary tremor, gait instability, dystonia, hypotonia, muscular rigidity and chorea [108]. These are often associated with “metabolic stroke”, which leads to bilateral focal brain lesions, characteristically in the globus pallidus externa and other regions of the basal ganglia, which occur in the absence of vascular stroke aetiologies [104, 106, 109–111]. Necrotic lesions in the basal ganglia often occur during or shortly after metabolic decompensation [84]. Nevertheless, in some reported cases, necrosis and CT/MR findings in patients do not occur with a history of severe metabolic acidosis [105]. Whilst the picture around the susceptibility of the basal ganglia is not fully illuminated, findings of bilateral lesions are consistent throughout differential diagnosis of organic acidurias and primary mitochondrial diseases. This may be related to the fact that basal ganglia and particularly the putamen and globus pallidus have a rich vasculature supply (middle cerebral artery, branching to lenticulostriate arteries) [112–114], an abundance of mitochondria [115–117] and the network as a whole is reported to demonstrate high metabolic activity and increased glucose and oxygen utilization [118–120]. Consistently, lacunar infarcts in the pars reticulata of the substantia nigra, a region functionally and histologically identical to the globus pallidus interna, have also been found in patients with MMA, supporting the idea that specific cell-types show particular susceptibility [121].

Finally, long-term neurological damage can be present in the visual and auditory systems. Especially, optic atrophy is an increasingly recognized late presentation of MMA, resulting in acute or chronic visual loss [107, 122]. Optic atrophy in MMA has been hypothesized to arise from mitochondrial dysfunction, due to the similarities in age at onset, presentation and progression between MMA and primary mitochondrial disorders, as well as the apparent beneficial effect of supplementation with coenzyme Q10 and α-tocopherol [79]. Sensorineural hearing loss is rare, with yet unclear etiology.

### Kidney damage

Kidney specific defects manifest as tubulointerstitial nephritis and renal tubular acidosis. End stage renal disease may also occur as early as the second decade of life. Most patients have evidence of mild tubular dysfunction during childhood, usually without overt signs of renal Fanconi syndrome, which may become very profound during episodes of metabolic decompensation that are usually triggered by infection, ultimately causing renal salt and bicarbonate losses. A study examining a cohort of 30 French patients found chronic kidney disease in 47% with a median onset of 6.8 years [123]. Similar results have been reported in a large cohort of 273 European patients, whose clinical evolution was related to the type of defect [124]. Proteinuria and hematuria are usually absent in most patients, and renal histopathology shows signs of tubulointerstitial nephritis both in humans and in animal models of MMA [125, 126]. Renal ultrasound usually shows poorly differentiated kidneys with decreased growth overtime [127]. The mechanisms of cellular toxicity, caused by accumulating organic acids and the mitochondrial defects, and its implication in the pathogenesis of kidney damage in MMA, are discussed in the section below.

#### Insights into mitochondrial disease pathways—the role of impaired mitophagy

Complete (*mut*^0^) or partial (*mut*^−^) loss of the enzyme MMUT leads to the accumulation of toxic organic acids (e.g., methylmalonic acid, propionic acid and 2-methylcitric acid) and loss of anaplerosis (Fig. 3, top panel). This presumably trigger the structural and functional abnormalities in the mitochondrial network (Fig. 3, bottom panel) which likely drive severe organ dysfunction affecting primarily brain and kidney. Reinforcing this concept, the metabolic signature of high urinary excretion of toxic organic acids, as well as mitochondrial morphological abnormalities, dysregulation of the respiratory chain complex, causing decreases in mitochondrial energy production, and augmented production of reactive oxygen species and oxidative damage have been described in kidney cells [126] and biopsies from MMA patients [128, 129]. However, mechanistically, how the enzyme deficiency begets mitochondrial distress and cell toxicity has remained incompletely understood.

**Fig. 4:**
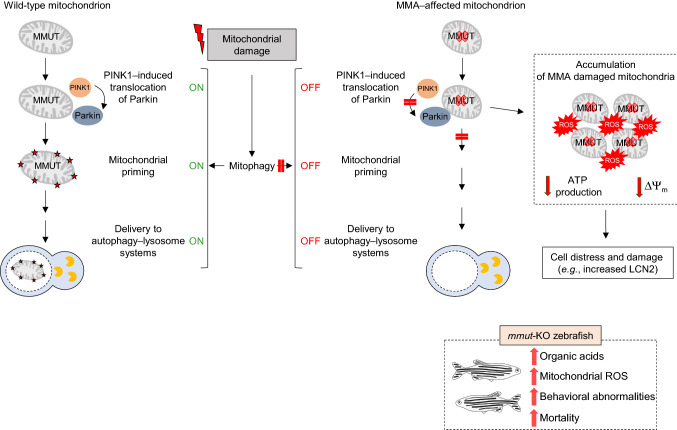
**Model depicting the link between MMUT, mitochondria, organelle quality control and surveillance systems, and epithelial homeostasis in normal and MMA‒affected kidney cells.** In MMA-affected kidney cells and zebrafish, deficiency of the enzyme MMUT and the resulting accumulation of toxic organic acids trigger mitochondrial alterations that are characterized by a collapse of the mitochondrial membrane potential (∆Ψm), abnormal bioenergetics profiling, and increased generation of mitochondrial ROS and oxidative stress. Faulty execution of PINK1-Parkin-mediated mitophagy induced by MMUT deficiency impedes the delivery of damaged mitochondria and their dismantling by autophagy-lysosome degradation systems. This leads to accumulation of damaged and/or dysfunctional, ROS-overproducing mitochondria that ultimately trigger cellular distress (as evidenced by Lcn2 overproduction) in patient-derived cells and disease-relevant phenotypes (as testified by liver/kidney mitochondriopathy, behavioral abnormalities and an excess of mortality) in *mmut-*deficient zebrafish (Adapted from Luciani and Devuyst [131])

Prolonged or unrepairable damage can lead to elimination of mitochondria through a selective autophagic process termed mitophagy. Given the accumulation of MMA-damaged mitochondria and autophagic vesicles, MMUT deficiency might compromise PINK1/Parkin-directed priming of MMA stressed mitochondria to autophagic-lysosomal degradation. Using the translocation of the protein parkin RBR E3 ubiquitin protein ligase (PRKN) to mitochondria as a *bona fide* reporter of PINK1-PRKN-dependent priming mechanisms, MMA patient-derived kidney cells display a decrease in numbers of PRKN^+^ clusters and translocation of PRKN to damaged mitochondria, in both normal and stress-induced conditions [130–132]. As a direct consequence of defective PINK1-PRKN priming mechanisms, mutant cells fail to deliver their damaged mitochondria to autophagy-lysosome degradation systems, causing the accumulation of dysfunctional mitochondria that trigger cellular distress and kidney damage [130–132]. Expression of the wild-type PINK1 in MMA patient-derived kidney cells activates mitophagy-mediated degradation of diseased mitochondria, thereby averting mitochondria-derived epithelial distress and cell damage. These findings suggest that anomalies in PINK1/Parkin-mediated quality control and surveillance systems might thus intersect the mitochondrial alterations induced by MMUT deficiency to reach a high level of mitochondrial dysfunction that ultimately contributes to the pathogenesis in MMA patients (Fig. 4). The concept that defective mitochondrial autophagy/mitophagy and uncontrolled cellular stress might contribute to the MMA disease is in line with the observed correlation between mitochondrial dysfunction, oxidative stress, and circulating lipocalin-2 (LCN2)—a secreted iron-transporting protein produced by kidney tubules following cellular damage in a cohort of patients with MMA [126, 129].

## Cell and animal-based disease models of MMA

### Patient-derived cells and tissue culture

Assessments of the mode of action of protein-damaging mutations require proof-of-concept studies in physiologically relevant cell culture based-systems. Traditional cell-based models typically rely on cancer-derived or immortalized cells that fail to adequately recapitulate the complex features of the in vivo disease phenotype [133]. The use of patient-derived primary cell samples aims thus to overwhelm the disadvantages and limitations of using transformed cell lines for dissecting disease mechanisms. These samples also provide more clinically relevant cell-based models for systematically testing drug discovery and screening pipelines or identifying novel drug-repositioning opportunities.

A common use of patient-derived cell lines in MMA is that of primary skin-derived fibroblasts for disease diagnosis. Although not a clinically important cell-type, fibroblasts express all proteins relevant to MMA. As such, they are routinely utilized for activity analysis of individual enzymes, particularly MMUT, or diagnosis of defects in the propionate pathway through incorporation of radio-labeled propionate into purified proteins [134]. To assess disease mechanisms, fibroblasts have been subjected to proteomics and metabolomics analysis, whereby dysregulation of serine metabolism was found [135]. A potentially more exciting use of patient-derived fibroblasts involves converting them to induced pluripotent stem cells (iPSCs), which can subsequently be differentiated into all cell types of the human body. At least two MMA patient-derived iPSC lines have been generated [136]. Differentiation of these, particularly into neural cells, may provide novel insights into CNS disease mechanisms, which are currently poorly understood.

Other potentially relevant patient-derived cell types are primary hepatocytes. Since the liver is a site for some clinical manifestations, including hepatomegaly [79] and potentially more concerning hepatocellular carcinoma [137] hepatocytes may be an important cell type to investigate. Using a novel organotypic system which utilizes hemodynamic flow, Collado and colleagues [138] demonstrated accumulation of disease relevant metabolites in patient-derived primary hepatocytes.

As MMUT is robustly expressed within the mitochondria of kidney tubular cells [130], we analyzed the properties of the mitochondrial network in kidney tubular cells derived from the urine of either healthy controls or *mut*^*0*^ MMA patients harboring inactivating mutations in *MMUT* (henceforward referred to as MMA cells).These studies revealed that MMUT deficiency causes accumulation of damaged and dysfunctional mitochondria, ultimately triggering cell distress and kidney damage [128, 130]. These dysfunctions are associated with an exaggerated production of LCN2—a small iron-transporting protein associated with kidney disease progression [139] and metabolic disease [140]. In line with recent studies stating dysregulation of autophagy-lysosome degradation pathways in mitochondria-related diseases [141] and in tissue samples from patients with MMA [129], autophagosome-associated LC3-II form, punctate LC3-positive structures, and electron microscopy structures compatible with autophagic vacuoles accumulate in MMA cells compared to their corresponding controls. Furthermore, MMA cells display decreased mitochondrial membrane potential and mitochondrial bioenergetics. These alterations are paralleled by a major mitochondrial oxidative stress [128, 130], as testified by the elevated production of mitochondria-derived ROS (MitoSOX, a live-cell permeant indicator of mitochondrial ROS), which are observed also in other mitochondria-related diseases [126, 141]. Small-molecules targeting oxidative stress have been shown to be efficacious in repairing mitochondrial homeostasis and function in MMA cells [130], demonstrating the utility of these cellular systems for translating disease knowledge to clinical outcomes for therapeutic approaches.

### Animal models

The availability of animal-based models represent a fundamental opportunity not only to gain insights into signaling frameworks driving the pathogenesis of rare genetic diseases but also to test potential therapeutic strategies.

Seven different mouse models of MMA have been generated to date, each with their own strengths. Two knock-out models of *Mmut* have been derived, resulting in complete null mutants [142, 143]. These mice displayed massive elevations of disease related metabolites, however, most pups did not survive past the first 24–48 h. Backcrossing to a mixed background did allow a small fraction of animals to survive the neonatal period [125]. These animals showed megamitochondria in hepatocytes, proximal tubule cells and exocrine pancreas, and displayed a clinical phenotype of tubulointerstitial renal disease. Ion-abrasion scanning electron microscopy from the liver of 4 day old *Mmut* knock-out mice confirmed the beginning of structural changes, but before conversion to megamitochondria [144]. Partial rescue was mediated by creating transgenic mice hemizygous for a human wild-type *MMUT* transgene [145] but not one incorporating p.Arg403* [146]. These mice were smaller than their littermates and had elevated methylmalonic acid in urine, plasma, and tissues. Full rescue has been achieved through expression of a stable transgene under the control of a liver-specific albumin promoter (Mut^−/−^;Tg^INS−Alb−Mut^, [126]) and a skeletal muscle specific murine creatine kinase promoter (Mut^−/−^;Tg^INS−MCK−Mut^, [129]). Nevertheless, Mut^−/−^;Tg^INS−Alb−Mut^ mice displayed chronic tubulointerstitial nephritis, had ultrastructural changes in the proximal tubule mitochondria, increased expression of Lcn2 [126] and severe kidney disease [147] while *Mmut*^−/−^;Tg^INS−MCK−Mut^ mice showed growth retardation, and liver and kidney mitochondriopathy [129]. A knock-in mouse [148] has been produced by Forny and colleagues which incorporated a missense change (p.Met698Lys in mouse) corresponding to a known patient mutation (p.Met700Lys in human). When present in the hemizygous form, along with a knock-out mutation on the second allele, these mice show a mild MMA phenotype which nevertheless includes elevated metabolites (methylmalonic acid, propionylcarnitine/acetylcarnitine ratio) early signs of renal dysfunction and TCA cycle alterations in liver mitochondria [149]. These symptoms are exacerbated in the presence of a high-protein diet, whereby mice exhibited a strong growth defect, morphological changes in the liver, low bone mineral density and ovarian atrophy [150]. Surprisingly, however, fasting for 24 or 48 h resulted in reduced metabolite levels [151].

A transgenic zebrafish model of MMA has been generated by using CRISPR/Cas9 genome editing [130]. The obtained zebrafish mutant line carries an 11-bp-CRISPR/Cas9-induced deletion (*mmut*^del11/de111^), generating a premature stop codon within exon 3, resulting in a truncated protein deprived of its catalytic activity. Analogous to metabolic and mitochondrial alterations reported in MMA patient-derived kidney cells, both liver and kidney of *mmut*‒deficient zebrafish accumulate methylmalonic acid and display altered mitochondrial morphology characterized by increased mitochondrial circularity with perturbed cristae organization. Metabolic flux analyses have revealed impaired mitochondrial bioenergetics in *mmut-*deficient zebrafish when compared to control larvae. These changes are paralleled by a major mitochondrial oxidative stress, suggesting an evolutionary conservation of this connection. Mutant zebrafish faithfully recapitulate MMA characteristic disease-relevant phenotypes, such as liver mitochondriopathy, behavioral abnormalities, and an excess of mortality. These phenotypes are rescued by feeding the mutant zebrafish with a low protein diet—a current supportive care strategy used in MMA patients [84]. Intriguingly, these findings revealed that restoring *mmut* activity in the liver, which normalizes the levels of methylmalonic acid metabolite and blunts excessive mortality, does not protect the *mmut-*deficient zebrafish from the abnormal swimming phenotype. The latter observation suggests that *mmut-*induced mitotoxicity in other cell types and organs (i.e., central nervous system, optic nerve and/or muscle) might govern the phenotypes encountered in *mmut-*deficient zebrafish. Given the faithful recapitulation of disease phenotypes associated with MMA, and the recent technological advances in scale, time, and cost, and multiplexing of conditions and the potential of automation, zebrafish might thus serve as a forefront tool for chemical phenotypic screens of “first in class” drugs, ultimately translating promising preclinical drug candidates into clinical success.

### Current therapies and future targetable strategies

Isolated MMA makes up one of the most frequent groups of inborn errors of metabolism, which often manifest in early childhood and are associated with high morbidity and mortality. There are no curative treatments for MMA. The available therapeutic approaches, such as dialysis and intravenous glucose infusion in the acute situation, as well as dietary protein restriction, supplementation with carnitine and vitamin B_12_ and potentially liver and/or kidney transplantation as long-term measures, aim for metabolic stabilization of the patient [84, 97]. While these measures can substantially decrease mortality and the overall morbidity, they cannot completely prevent long-term complications. Therefore, there is an urgent need to yield promising targetable interventions in the early course of MMA.

Previous studies showed that kidney dysfunction and levels of circulating Lcn2 could be abrogated in a transgenic mouse model of MMA by administrating ubiquinone, a bioavailable form of coenzyme Q_10_ that acts on mitochondria, and vitamin E [126]. Taking advantage of a drug-disease network-based computational modeling approach (Mantra 2.0; Mode of Action by Network Analysis; http://mantra.tigem.it), we identified new drug-able pathways that might potentially counteract cellular dysfunctions associated with MMA [130]. These in silico analyses indicated that targeting mitochondrial function and redox homeostasis might reverse disease phenotypes associated with MMUT deficiency. In line with these predictions, treatment of MMA cells with mitochondria-targeted antioxidants such as mito-TEMPO partially rescues mitochondrial network homeostasis and improves its function, blunts any increases in mitochondrial ROS and autophagy markers, and prevents epithelial distress and cell damage [130]. In a similar vein, treatment with low doses of MitoQ, another well-known mitochondria-targeted antioxidant, alleviates the mitochondrial oxidative stress, improves behavioral phenotypes, and reduces the excessive mortality in the *mmut-*deficient zebrafish model of MMA [130]. Importantly, both pharmacological interventions did not modify the levels of MMA metabolite in either MMA cells or *mmut*‒deficient zebrafish, supporting the concept that mitochondrial targeting acts independently of the elevation of toxic MMA metabolites. Ultimately, regimens that enhance PINK1-Parkin-directed mitophagy and mitochondrial quality control might facilitate the degradation of MMA-affected mitochondria and attenuate the cellular and metabolic alterations that drive life-threatening systemic manifestations in MMA patients. In this case, the administration of either synthetic or natural chemical compounds with direct or indirect mitophagy-augmenting properties [e.g., NAD^+^ precursors, such as nicotinamide riboside (NR), nicotinamide mononucleotide (NMN) and nicotinamide (NAM) (reviewed in [11]); autophagy and mitophagy activators such as spermidine [152], resveratrol [153], urolithin A [34]; mitochondrial stress response inducers such as actinonin [154] and doxycycline [44] would thus constitute an attractive (and potentially feasible) strategy for therapeutically treating this devastating disorder.

Alternatively, using primary hepatocyte disease models, a group of scientists from HemoShear Therapeutics have shown that supplementation with disodium citrate partially rebalances the concentration of TCA cycle intermediates [138], while supplementation with a short-chain carboxylic acid (2,2-dimethylbutanoic acid) is able to reduce intracellular concentrations of metabolites related to propionyl-CoA and methylmalonyl-CoA [155, 156]. This latter molecule is now part of a therapeutic clinical trial (NCT04732429) for MMA.

Beyond promising candidate targets for drug development, cell-based therapy including the direct replacement of defective MMUT through the administration of gene or mRNA therapy has been tested in various mouse models of MMA. In a first attempt, adenovirus mediated gene therapy of *Mmut* under the control of a cytomegalovirus promoter delivered either intramuscularly or intrahepatic demonstrated that half of the *Mmut* knock-out mice provided with intrahepatic injections, which would otherwise die within 48 h postnatal, survived past weaning [157]. Later, treatment with adeno-associated virus (AAV) serotype-8 resulted in much longer survival of *Mmut-*KO mice [158, 159], while injection with *Mmut* under a liver-specific thyroxine-binding globulin promoter resulted long-term phenotypic correction following administration with AAV8 [160], or AAV9 [161]. Further liver directed therapies have included lentiviral administration of a codon optimized human *MMUT* transgene, which resulted in correction of the growth defect, and reduced methylmalonic acid in plasma, urine and tissues [162]. Most recently, a promoterless *MMUT* transgene has been delivered into the albumin locus by AAV, resulting in improved animal survival and an apparent growth advantage to correct hepatocytes [163]. Finally, using non-viral delivery, lipid nanoparticle enclosed human *MMUT* mRNA delivered via tail vein injection and targeting the liver has improved survival and weight gain and reduced circulating metabolites in *Mmut*^−/−^;Tg^INS−MCK−Mut^ and *Mmut*^−/−^;Tg^INS−CBA−G715V^ mice [164, 165]. Both AAV-mediated gene therapy using promoterless *MMUT* (NCT04581785) and LNP-mediated mRNA therapy (NCT03810690) are the focus of ongoing phase 1/2 clinical trials.

## Conclusions

The maintenance of a healthy mitochondrial network is particularly crucial for cellular and organismal homeostasis, and loss-of-function mutations that impair the function of mitochondria can invariably confer a potentially devastating vulnerability to many different cell types, ultimately contributing to a broad spectrum of diseases. Inherited defects in mitochondrial-localized proteins and/or enzymes, as exemplified here by MMA, might disable PINK1/Parkin-mediated quality control and surveillance systems, triggering a level of mitochondrial dysfunction that drives cellular distress and tissue damage.

The mitochondrial dysfunction and impaired mitophagy flux seem to play a pivotal role in the pathogenesis of kidney damage, a so far poorly understood process, and, possibly, also in the generation of central nervous system-related symptoms and of neuropathological signs in MMA patients. Interestingly, and unlike cardiomyocytes of rodents lacking *Pink1* [166] or *Parkin* [167], which display accumulation of morphologically abnormal mitochondria and heart pathology, patients with MMA rarely manifest symptoms of cardiomyopathy [84, 101]. The reasons for this exquisite context and cell type specificity are fundamentally enigmatic. Tentative hypotheses, which warrant further investigations in non-affected tissues, include the existence of compensatory (stress-evoked) surveillance systems that coordinate the mitochondrial turnover and quality control, or the cumulative effect of the metabolic perturbations resulting from the absence of MMUT enzyme and the loss of PINK1/Parkin-mediated mitophagy to reach a high level of mitochondrial dysfunction that ultimately causes disease relevant-phenotypes in MMA. Indeed, the accumulation of methylmalonic acid in the blood of older people seems to sustain age-induced spread of cancer [168]—an emerging complication encountered in patients with MMA [169].

The mechanisms by which MMUT deficiency suppresses PINK1/Parkin-directed “eat me” signals and hence mitophagy, remain equally elusive. An intriguing scenario might be that MMUT deficiency might alter the stability of PINK1 by disabling the interaction with yet-to-be defined factors that protect PINK1 from processing and degradation. Alternatively, MMUT deficiency might trigger stress-related posttranslational modifications such as S-nitrosylation that inhibits PINK1 kinase activity and hence mitophagy-directed degradation pathways [168]. We suspect that dysregulation of adaptive response to mitochondrial stress might also contribute to maladaptation and disease in patients with MMA, and this will require further studies to understand the effects of *MMUT* mutations on mitochondrial repair pathways, such as UPR^mt^ and mitochondrial biogenesis. These questions are just examples of all the exciting work that lies ahead to comprehensively dissect the cell type-specific functions of MMUT and mitochondrial quality control systems. A current challenge is to translate the knowledge gained from fundamental studies of mitochondrion biology to the treatment of MMA and other mitochondria-related diseases. In this regard, the use of informative preclinical models and physiologically relevant cellular systems, coupled with improved knowledge of cell biology-disease signatures and the recent advances in multi-omics technologies, might accelerate the development of therapeutics that can halt the progression of MMA disease as well as other rare and more common diseases associated with mitochondrial dysfunction.

## Data Availability

Not applicable.
